# Supplementary material for: Age-differentiated incentives for adaptive behavior during epidemics produce oscillatory and chaotic dynamics
Source: PLoS Comput Biol. 2023 Sep 5;19(9):e1011217. doi: 10.1371/journal.pcbi.1011217 (PMC10503720; doi:10.1371/journal.pcbi.1011217)
Supplement: Attachment — Submitted filename: Pcompbiol-d-23-00840_response_MF.docx [file pcbi.1011217.s001.docx]

**Response to Review of Manuscript # PCOMPBIOL-D-23-00840**

Received: June 26, 2023

Please note we have changed the title from “Age classes stratified by risk and adaptive behavior during epidemics” to “Age-differentiated incentives for adaptive behavior during epidemics produce oscillatory and chaotic dynamics” and modified the last four lines of the abstract.

Responses to Reviewers’ Comments and Questions

**Comments to the Authors:
Please note here if the review is uploaded as an attachment.**

**Reviewer #1:** The manuscript discusses how adaptive behavior can cause the emergence of chaos in an SIS model. The results are interesting and the manuscript is well-written, but I believe some further analysis is necessary for publication. In particular, for the utility function (equation (2.4)) a robustness analysis would be interesting. What happens if the cost function has a different form?

*Response. The utility function was originally developed in a previously-published paper (Arthur et al., 2021). This function represents a tradeoff between the economic costs of reducing social contact rates and the public health costs of increasing social contact rates during an epidemic. The cost of deviating from a target is modeled in quadratic form, consistent with engineering, economic, and statistical modeling (take e.g. the least squares regression technique, which looks at deviations from the model, or target, and squares the “error”). This quadratic form yields a concave function with mathematically-appealing properties, which is why it’s typically used in this way. If the deviation were assumed to be linear (i.e.,* $c-\hat{c}$*), the differentiation of the utility function to maximize for U would eliminate the* $\hat{c}$ *term and the relationship between* $\hat{c}$ *and c would thus become meaningless. The functional form could be cubic, for example, but there is little rationale or empirical evidence to suggest that the penalty for deviating from the best contact rate economically should have such a polynomial form.*

*Other potential functional forms for how adaptive behavior is modulated by epidemic features, such as prevalence, are adopted by other modelers in other papers. For some examples, Capasso and Serio (1978); Glaubitz and Fu (2020); Harris et al. (2023); Reluga (2010), and others. Many of these do find oscillatory dynamics in their results, but they do not generally find chaotic attractors in resulting dynamics, though ecological theory has found these dynamics. Negative feedbacks with time delays will naturally produce oscillations, so this is not generally surprising. And the economic epidemiology of this was worked out by Philippson (2000). These have been added to the list of references.*

*We have added more on the model at Lines 69-71 and Lines 136-139.*

Additionally, the Figures 2 and 4 need thicker lines to be legible. For Figures 1,3,5 and 7, the text needs to be larger.

*Response. Figs 2 and 4 contain points, rather than lines. We have experimented with larger points, but the points then blend together and the information provided by the bifurcation diagrams is reduced. Figs 1,3,5,7 now have larger text.*

**Reviewer #2:** This is a relatively novel article that explores the impact of heterogeneity in exposure patterns, mortality, and transmissibility among different age groups on epidemic outcomes. By changing the propagation rate and the contact parameters between age groups, it is well demonstrated that the adaptive behavior may cause the system to converge to non-zero equilibrium, and the behavior of period-doubling bifurcation, chaos and collapse may occur with the increase of parameters. I would like to recommend the paper for publication once the following problems are well addressed.
1. The classification of the three age groups is a little vague, and it is best to have a specific age range for the elderly, which needs to be explained.

*Response. Specific age ranges are now given in the last paragraph of the introduction, consistent with Acemoglu et al. (2021) and the empirical evidence for differentiated mortality by age. Lines 69-71 now read: ‘Here, we model an infectious disease with adaptive behavior incentives of the form used in a previous model and include different mortality rates for three age classes: the “young" (ages 20-49), the “middle-aged" (50-64), and the “old” (65+) (a la Acemoglu et al. (2021)’.*

2. The incubation state E is not considered, which may not be infectious. Whether it is necessary to add E state to the model needs to be explained separately.

*Response. The incubation state E is indeed not modeled here, nor is the recovered state R. It is our objective to model an infectious disease with age-differentiated behavior as simply as possible, rather than to use a model that is tailored for a particular infectious disease. We have included the following in the limitations paragraph in Lines 191-195:*

*‘First, the model is of the SIS type and does not consider an Exposed class (E) or a Recovered class (R). The inclusion of E or R (in e.g. SEIRS models) may decrease the number of susceptible individuals and thus slow some of the oscillatory or chaotic dynamics found here. The inclusion of R for SIR models depletes susceptibles and would prevent sustained dynamic patterns.’*

3. The recovery rate from the infected category to the susceptible is the same for all age classes, namely, for all i, j. Whether it is a little unrealistic, the recovery time of older people should be longer than that of younger people.

*Response. Indeed, the constraints listed in Eqns 2.10-2.12 could be made more realistic. Differences in the age groups may be identified in other epidemiologically-relevant aspects as well. However, the model’s age differentiation is inspired by COVID-19, but the model is not parameterized to COVID-19. These assumptions were adopted to simplify the model and isolate the parameters of interest for aversion to infection and target contact rates. Heterogeneity in gamma parameters would also make for an interesting extension of this model.*

4. Figure 1. Why does the higher the transmission rate b, the more it tends to collapse? It is better to explain it in the paper.

*Response. The epidemiological parameter R*_0_*, the reproduction number, is directly proportional to the transmissibility of a particular infectious disease and to the contact rate, so the higher the b*_0_ *parameter or the c_ij_ parameter, the higher the R*_0_*. The reproduction number is analogous to a growth rate in population dynamics or ecology. Our results here are directly comparable to the well-studied logistic map, meant to represent the population dynamics of a single species in a resource-limited setting (Ausloos, 2006). In the logistic map, the resulting dynamic regimes of collapse, monotonic convergence, damped oscillators, oscillators, period doubling, chaos, and collapse all depend on the value of the growth rate r, and particular thresholds have been identified for r that result in regime shifts.*

*The following (Lines 241-247) has been added to Paragraph 5 of the discussion, which looks at the relationship between our epi model and ecological models:*

*‘For example, the logistic map is an extensively-studied simple model of population growth with limited resources that exhibits similar dynamics to our adaptive behavior model: convergence, oscillations, chaos, and collapse (Ausloos, 2006). The driver of these dynamics in the logistic map is r, the growth rate. In our model, two of the principal drivers are b*_0_*, the transmissibility, and c_ij_, the between-group contact rate, as the epidemic’s reproduction number R*_0_ *is directly proportional to transmissibility and contact rate.’*

5. The explanation in Figure 5 is short of what needs to be explained. Are there any meaningful findings?

*Response. Some additional text has been added to the caption of Figure 6 (formerly Fig 5) to help guide the reader: ‘Dynamic regimes are indicated by color and each graphic increases transmissibility.’*

*The following has been added to the Results section in Lines 153-157:*

*‘The aversion to infection for the elderly and the youth given by* $\alpha_{22}$ *and* $\alpha_{21}$*, respectively, may produce more complex dynamics at higher values, but the relationship between the two values is also important (Fig 5). Simpler dynamics (e.g., equilibrium and 2-point cycles) can be found closer to the line* $y=x$ *(i.e.,* $\alpha_{22}=\alpha_{21}$*), while highly differentiated aversions to infection are more likely to produce chaos or oscillations of higher periodicity.’*

*The following has been added to the Discussion section in the paragraph on the relationship between the youth and elderly in Lines 217-219:*

*‘Indeed, even at lower levels of infection aversion for the elderly, the more differentiated the aversions to infection between the two populations, the more complex the dynamics (Fig 6).’*

6. Why with the increase of b, there will be a collapse phenomenon, the article needs to analyze the reason for this phenomenon.

*Response. As above, b*_0_ *is analogous to r in the logistic map and other mathematical models that investigate chaos. Collapse (or divergence out of the bounds of the system) is a common finding from these models.*

7. In the figure, infected should be explained. Is it a daily infection? Although it is reflected in the figure, it should be explained to distinguish it from cumulative infection. In addition, it should be explained why infected people oscillate around a certain value. Why is it stable around some value?

*Response. Infected represents the number of individuals in the infected class at any given time. Captions in Figs. 1 and 3 have been updated to explicitly state ‘number of infected individuals over time,’ rather than ‘disease dynamics.’ Captions in Figs. 2 and 4 have been expanded: ‘Points in the figure represent sustained long-term results in the model, thus any* $b_{0}$ *value with, for example, two corresponding y-axis points represents a sustained oscillation between 2 values for infected individuals.’*

*Stable oscillatory patterns are a function of chaotic systems with negative feedback mechanisms. More attention to and explanation of the oscillations is provided in Arthur et al. (2021).*

8. The innovation of the article needs to be highlighted, what is the difference from the previous work, or what is the extension of the work of others.

*Response. The innovation of this article is the inclusion of age structure into an adaptive behavior model with heterogeneous aversion to infection and contact patterns.*

*The abstract has been modified (lines 12–15) and the following sentence has been included to open the discussion, Lines 178-179:*

*‘Our model has generalized previous work on adaptive behavior during epidemics to include age structure with heterogeneous aversion to infection and target contact rates.’*

9. In Chapter 4, why are the parameters set this way in the numerical simulation, and what needs to be introduced.

*Response. The following has been added to introduce the default parameters on Lines 136-139: ‘No empirical research has been done to analyze comparative utility lost or gained as a function of contacts. Thus, the parameter set has been chosen to be demonstrative of possible dynamic regimes, rather than parameterized to COVID-19 or other infectious diseases. These default parameters are:’*

10. Where in lines 186-187 does the influence of young people on old people's transmission need to be explained.

*Response. Yes, to further explain Figure 6 (formerly Fig 5) we include a new figure 5 that demonstrates that homogeneity with the same parameters but no interactions produces simpler dynamics and bolsters the argument that how the youth act impacts the elderly.*

*In addition to the above in response to Point 5, the following sentence has been added to Paragraph 3 of the discussion on Lines 212-213:*

*‘Without contact with other groups, the Elderly age class exhibits simpler dynamics (e.g. monotonic convergence) (Fig 5).’*

11. Since there is oscillating behavior between different age groups, is there also this period-doubling bifurcation behavior for the whole population N? You can also make a graph to illustrate that it is best to compare the oscillating behavior between the whole population of age group 2 and age group 3 with basically the same parameters.

*Response. Yes, when the population is homogeneous, many of these dynamic regimes are still present as studied in Arthur et al. (2021). To investigate the impact of the interactivity on homogeneous systems with the same parameters, we have created a new figure that shows the dynamics of the 2-population system without interaction (Fig 5). From this exercise, we see that the effect of heterogeneous interaction is to exhibit more chaotic dynamics at lower levels of b*_0_ *when the two populations interact and for the elderly dynamics to especially increase in complexity.*

*We have included the following text in the Results section in Lines 148-152:*

*‘When* $c_{12}=0$ *and* $c_{t}^{*}$ *is only based on prevalence in the same age category, we see what would happen in a homogeneous population without age structure with the same parameters (Fig 5). Without this interdependent age-structure, Youth dynamics are even more complex, while Elderly dynamics have less complexity.’*

Reviewer #3: The authors place attention on the effects of social and behavioral factors in driving the dynamics of infectious diseases, primarily examining contact mixing and severity-based aversion to infection. It is nice to see these matters incorporated into modeling, especially as willingness to engage in risk-mitigating behaviors was a highly contentious matter while attempting to control COVID-19 spread in the United States.

Specific comments:

1. Lines 94-97: I understand the need to avoid optimizing all elements of a contact matrix, but does this impose any limitations on the model, such as needing to define groups such that mixing will not change substantially during an epidemic? This is reasonable for the age groups selected by the authors (children, working-age adults, elderly adults), but would be more difficult to justify if using finer age groups, such as 10-year age bands.

*Response. Yes, we agree with this assessment. We view setting between-group contact rates as fixed and within-group contact rates as adaptive as a reasonable simplifying assumption, but we recognize this assumption in the limitations section. Further, we have added this sentence in the limitations section in Lines 202-204:*

*‘Expanding this model to finer age categories than the ones used in this paper should include empirically-derived contact rates and differentiated optimization for between-group contacts.’*

2. Lines 134-135: How were these parameters selected? It would be beneficial if these values were chosen based on realistic resemblance to existing infectious diseases, whether through the parameter values themselves or by the disease dynamics produced through this model parameterization.

*Response. Yes, this is true. Given the novelty of the formulation of this model and the lack of empirical research investigating adaptive behavior and its utility during epidemics, we cannot parameterize our model for a particular infectious disease. We thus do not know if COVID-19 or another infectious disease are within the parameter ranges to produce equilibria, oscillations, or chaos, we simply know that these dynamic regimes exist in theory. However, given the empirical evidence from COVID-19, oscillations certainly did occur in multiple geographies over multiple timeframes, some of which may have been due to adaptive behavior and heterogeneous age structure. And some evidence has been provided that indicate COVID-19 may have had chaotic dynamics as well (Sapkota et al., 2021).*

We have added the following to the limitations paragraph in Lines 206-211:

*‘We note the model was not trained on real-world data to produce parameters as empirical data on adaptive behavior and incentives is lacking. While the dynamical regimes found here exist in theory, we do not know where in practice real-world disease systems may be. However, COVID-19 exhibited oscillatory patterns and chaotic dynamics, suggesting that this theoretical work may be directly relevant to diseases with strong adaptive behavior components.’*

3. Similarly, it would be good to see if the results shown in Figures 1-6 are robust to other sets of baseline parameters. As the authors adjusted single or pairs of parameters, the others remained at these default values. Had different default values been chosen, would there be notable differences in the results?

*Response. Yes, with different default parameters, this transition between dynamic regimes is always possible. We deliberately chose parameters to demonstrate the regime types, rather than selecting parameter sets a priori to report resulting dynamics. We have added a figure to examine the inclusion of time-delay into the results and included the following in Lines 175-176:*

*‘When a time-delay is introduced (*$\Delta=1$*), the dynamics become complex at smaller levels of b*_0_ *(Fig 9) than when* $\Delta=0$ *(Fig 7).’*

4. This work presents how an SIS model is affected by social behaviors. However, it is common for infectious pathogens to confer at least temporary immunity following infection. While I recognize that extending all of the methods/results for an SIRS model could be unnecessarily complex and for little gain, it would be worthwhile in the discussion to describe how other model setups beyond an SIS model would likely be affected by the social and behavioral factors from this study.

*Response. Yes, we have added the following to the limitations paragraph in the discussion in Lines 191-195:*

*‘First, the model is of the SIS type and does not consider an Exposed class (E) or a Recovered class (R). The inclusion of E or R (in e.g. SEIRS models) may decrease the number of susceptible individuals and thus slow some of the oscillatory or chaotic dynamics found here. The inclusion of R for SIR models depletes susceptibles and prevents sustained dynamic patterns.’*

**Have the authors made all data and (if applicable) computational code underlying the findings in their manuscript fully available?**
The [PLOS Data policy](https://journals.plos.org/ploscompbiol/s/materials-and-software-sharing) requires authors to make all data and code underlying the findings described in their manuscript fully available without restriction, with rare exception (please refer to the Data Availability Statement in the manuscript PDF file). The data and code should be provided as part of the manuscript or its supporting information, or deposited to a public repository. For example, in addition to summary statistics, the data points behind means, medians and variance measures should be available. If there are restrictions on publicly sharing data or code —e.g. participant privacy or use of data from a third party—those must be specified.

Reviewer #1: None

Reviewer #2: None

Reviewer #3: **No:**

PLOS authors have the option to publish the peer review history of their article ([what does this mean?](https://journals.plos.org/ploscompbiol/s/editorial-and-peer-review-process#loc-peer-review-history)). If published, this will include your full peer review and any attached files.

If you choose “no”, your identity will remain anonymous but your review may still be made public.

**Do you want your identity to be public for this peer review?** For information about this choice, including consent withdrawal, please see our [Privacy Policy](https://www.plos.org/privacy-policy).

Reviewer #1: No

Reviewer #2: No

Reviewer #3: No

**References added in the revised manuscript**

Arthur, Ronan F., et al. "Adaptive social contact rates induce complex dynamics during epidemics." *PLoS computational biology* 17.2 (2021): e1008639.

Ausloos, Marcel. *The logistic map and the route to chaos: From the beginnings to modern applications*. Springer Science & Business Media, 2006.

Capasso, Vincenzo, and Gabriella Serio. "A generalization of the Kermack-McKendrick deterministic epidemic model." *Mathematical biosciences* 42.1-2 (1978): 43-61.

Glaubitz, Alina, and Feng Fu. "Oscillatory dynamics in the dilemma of social distancing." *Proceedings of the Royal Society A* 476.2243 (2020): 20200686.

Harris, Mallory J., Kimberly J. Cardenas, and Erin A. Mordecai. "Social divisions and risk perception drive divergent epidemics and large later waves." *Evolutionary Human Sciences* 5 (2023): e8.

Philipson, Tomas. "Economic epidemiology and infectious diseases." *Handbook of health economics* 1 (2000): 1761-1799.

Reluga, Timothy C. "Game theory of social distancing in response to an epidemic." *PLoS computational biology* 6.5 (2010): e1000793.

Sapkota, Nabin, et al. "The chaotic behavior of the spread of infection during the COVID-19 pandemic in the United States and globally." *Ieee Access* 9 (2021): 80692-80702.
